# Strengthening the WHO Emergency Care Systems Framework: insights from an integrated, patient-centered approach in the Copenhagen Emergency Medical Services system—a qualitative system analysis

**DOI:** 10.1186/s12913-025-12465-7

**Published:** 2025-03-18

**Authors:** Simone Böbel, Jeske Verhoeven, Mirjam Scholz, Bart Penders, Lorraine Frisina Doetter, Helle Collatz Christensen, Thomas Krafft

**Affiliations:** 1 Department of Health, Ethics and Society, Care and Public Health Research Institute (CAPHRI), Faculty of Health, Medicine and Life Sciences, Maastricht University, Maastricht, Netherlands; 2 Fraunhofer Institute for Manufacturing Engineering and Automation IPA, Stuttgart, Germany; 3 Käte Hamburger Kolleg “Cultures of Research” (Core), RWTH Aachen University, Aachen, Germany; 4 Collaborative Research Centre (CRC) 1342 & Research Center on Inequality and Social Policy (SOCIUM), The University of Bremen, Bremen, Germany; 5 Prehospital Center, Region Zealand, Næstved, Denmark; 6 Department of Clinical Medicine, Faculty of Health and Medical Sciences, University of Copenhagen, Copenhagen, Denmark

**Keywords:** Prehospital, Emergency medical services, Copenhagen, WHO emergency care system framework, Integrated care, Patient-centered care, Smart technology

## Abstract

**Background:**

The World Health Organization Emergency Care Systems Framework (WHO ECSF) was designed to offer guidance in establishing and developing effective Emergency Medical Services (EMS) systems. However, evolving disease patterns, changing community needs, and a rising demand for emergency care services, highlight the need for more integrated and patient-centered EMS systems. This evolution should be mirrored in the WHO ECSF. Hence, this study explores system components of the Copenhagen (CPH) EMS that may enhance the WHO ECSF´s emphasis on integrated and patient-centered care.

**Methods:**

A qualitative case study was conducted from April through June 2021, including (i) semi-structured interviews with researchers and professionals at the CPH EMS and (ii) a scoping literature review using PubMed, Google Scholar, expert recommendations and snowballing.

**Results:**

Thirteen expert interviews and 35 records were analyzed, revealing key integrated care components within the CPH EMS. These include education and citizen participation programs, early triaging, differentiated care pathways coordinated with primary care and out-of-hours services, and specialized mobile care units complementing “traditional” ambulance services. Technology supports integrated and patient-centered care by facilitating early differentiation of care, efficient dispatching, and communication. Data-driven approaches were fostered through technology-aided data collection, supporting research, quality improvement, and patient safety. The identified components were mapped within the WHO ECSF´s four domains: scene, transport, facility, and cross-cutting elements. Due to the prehospital focus of the CPH EMS, limited data was available for the “facility” site.

**Conclusions:**

The CPH EMS demonstrates an integrated, patient-centered systems approach that emphasizes seamless coordination along the patient care pathway, bridging EMS with broader health and social systems. Research-informed initiatives and intelligent technology solutions underscore the potential for enhancing the WHO ECSF. These findings highlight the importance of continued system integration and a holistic health perspective, including in emergency settings. Further research is needed to assess the transferability of these components across diverse global contexts.

**Trial registration:**

Not applicable.

**Supplementary Information:**

The online version contains supplementary material available at 10.1186/s12913-025-12465-7.

## Background

The demand for emergency medical care is increasing globally [1–3]. As disease patterns evolve and demographic and socio-cultural structures shift [2, 4–10], Emergency Medical Services (EMS) systems are challenged to meet increasingly diverse and complex patient needs with timely, appropriate care. However, often encountered patchworks of definitions, legislations, and health system structures [11–13], continue to cause fragmented and siloed structures across multiple regions or countries, complicating efforts to direct patients to the most suitable care pathway [14, 15]. Troubled by high numbers of Emergency Department (ED) visits, long waiting times and financial losses due to mismanagement of healthcare allocation, some countries in Europe have started to reconfigure urgent and emergency EMS systems towards a more integrated approach [16–20]. Similarly, the 76th World Health Assembly emphasizes the need for seamless coordination between emergency, critical, and primary care through effective communication, transport, and referral systems. As interdependent parts of the wider health system, failures in emergency care capacity disrupt primary care, while gaps in primary and social services increase emergency demand, potentially delaying life-saving care [21].

**Fig. 1:**
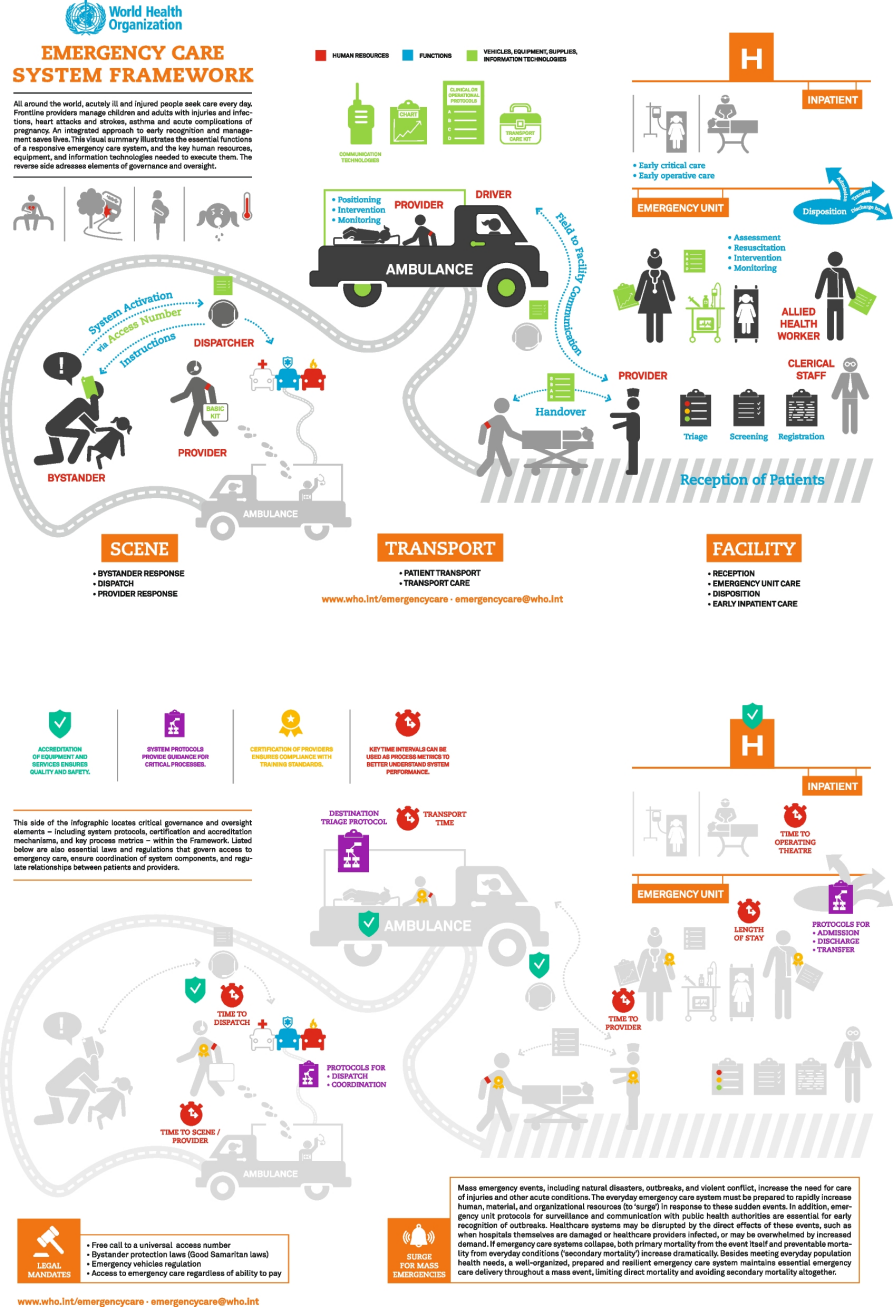
WHO Emergency Care System Framework (2018) [22]

The WHO Emergency Care System Framework (ECSF) was developed to provide a structured approach for organizing emergency care from initial contact through inpatient treatment (Fig. 1), aiming to support policymakers to evaluate and strengthen emergency care systems [22]. Despite this guidance, the ECSF largely emphasizes traditional hospital-based care pathways, which may not fully address the needs of patients with non-urgent or complex health issues. Thus, evidence-based insights are needed on how these systems can evolve to address shifting demands effectively. To our knowledge, an assessment or analysis of the completeness, currency, or adaptability of the WHO ECSF has not yet been published, highlighting an important gap that this study seeks to address by reviewing the Emergency Medical Services in the Capital Region of Denmark “*Hovedstadens Akutberedskab”* (CPH EMS) against the WHO ECSF.

Over the past two decades, CPH EMS has undergone a profound transformation, evolving from a fragmented and complex system into an integrated model within the broader healthcare framework. This integration now enables seamless coordination with primary care, out-of-hours services (OOH), and other emergency response entities, such as police and fire services [23–26]. By 2020, six years after its transformation, CPH EMS demonstrated significant performance improvements while reducing overall costs, showcasing the efficiency and sustainability of the restructured EMS framework [27]. ED waiting times reached historically low levels, while ED visits decreased by 10% within the first years following the system overhaul. The number of home visits by General Practitioners (GPs) also declined. Call response times were short, with emergency calls answered within 4–5 s and non-emergency calls within less than three minutes. Patient satisfaction reached 90%, with complaints averaging just 15 per month per 100,000 calls. Patient safety incidents were rare, with a thorough follow-up conducted on every case daily [27].

Enabled by Denmark's unique civil registration number system, comprehensive health data collection and linkage across registers [28–30], CPH EMS exemplifies the potential of research-driven innovation in EMS systems across Europe [31]. Additionally, through international initiatives such as co-founding the European EMS Leadership Network which addresses EMS challenges and innovations across Europe [32], and the Global Resuscitation Alliance (GRA), dedicated to advancing resuscitation practices [33] CPH EMS has played a key role in shaping the field. CPH EMS has also initiated, hosted, and co-organized European EMS Congresses [34], further strengthening international collaboration.

Thus, the CPH EMS, with its rapid transition towards an integrated, and patient-centered system and its emphasis on research-driven innovations, and international network and outreach is believed to serve as one good case in point for further advancing the WHO ECSF.

## Methods

### Aim

This study aims to analyze key components of the CPH EMS to inform potential enhancements to the WHO ECSF using a scoping review and expert interviews. Specifically, this study sought to:

1. Identify CPH EMS key components exemplifying its integrated and patient-centered approach.
2. Highlight elements within CPH EMS that could inform potential enhancement of the WHO ECSF.

By focusing on integrative, patient-centered practices and evolving evidence-driven EMS approaches, this qualitative analysis supports a practical review of the WHO ECSF. Rather than comprehensively reviewing the CPH EMS or fully updating the WHO ECSF this highlights the importance of continuous evaluation of the WHO ECSF.

### Study design

This exploratory study conducted a partial system analysis of the CPH EMS, using a qualitative approach [35] conducting expert interviews and a scoping review.

The initial step involved a partial system analysis, comparing the CPH EMS system with the WHO ECSF [36]. Given resource limitations, the analysis centered on selected examples of components based on the WHO Health System Building Blocks, rather than a full system analysis as proposed in the PEMS assessment tool by Mehmood et al. [37]. Data collection and analysis were performed in a concurrent manner, with analytic steps guiding additional data collection, and data initiating new analytic processes [38].

To ensure transparent and comprehensive reporting, the PRISMA ScR-Checklist [39] and COREQ-Checklist (COnsolidated criteria for REporting Qualitative research) [40] were followed in the reporting of this study.

### Setting: pre-hospital EMS of the capital region of Denmark

Established in 2011, CPH EMS serves 1.9 million (approximately one-third of Denmark´s population) across rural and urban areas spanning 2,563 km^2^ [23, 41], coordinating emergency medical communications, dispatch, mobile care units, and manages the region's overall interdisciplinary healthcare response and Major Incident Medical Management, including outbreak and preparedness planning [23, 42, 43]. Per year, the CPH EMS responds to 130.000 emergency medical calls (112), 1.2 million medical helpline calls (1813), and responds with 300.000 emergency ambulance missions and 500.000 non-urgent patient transports [27].

**Fig. 2:**
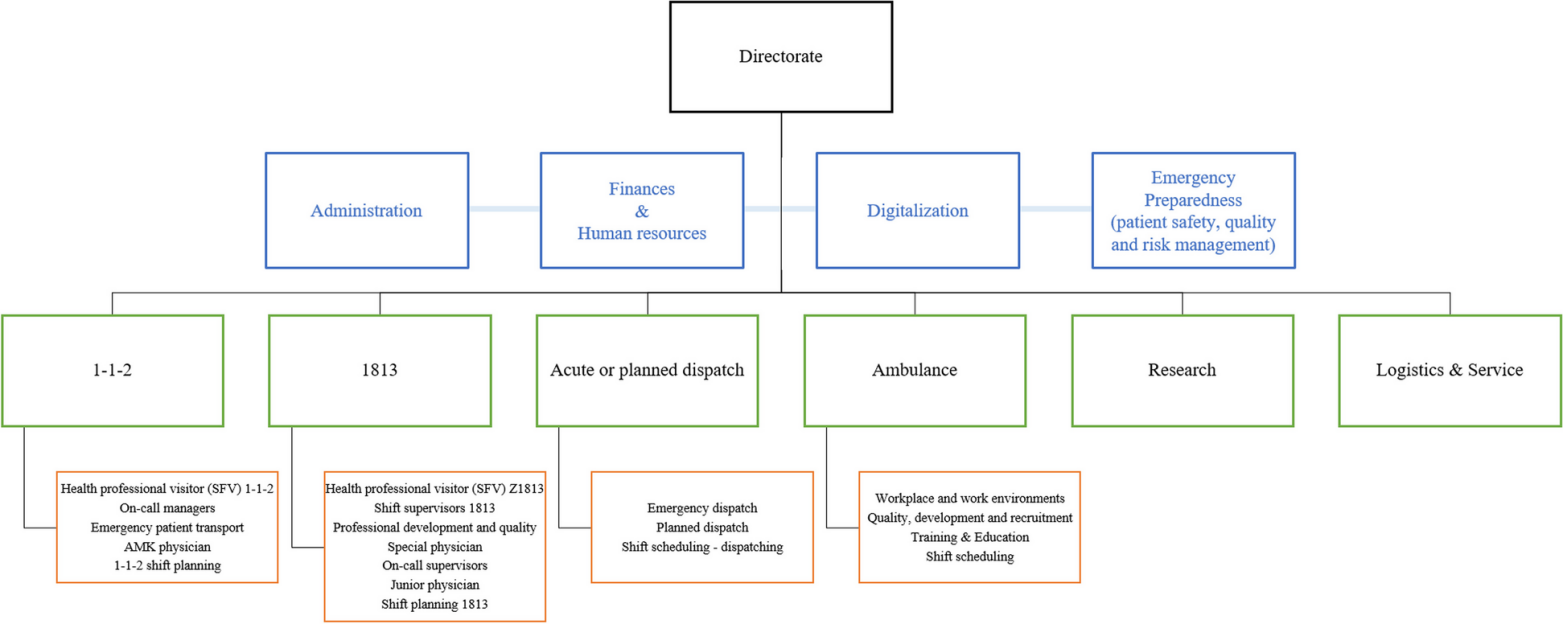
Organizational structure of the Copenhagen Emergency Medical Services (amended and translated from [46])

In Denmark´s tax-funded health system, the EMS operates as an independent Public Health organization responsible for acute and prehospital EMS within a national structure of five administrative and health care regions, each responsible for health and psychiatric services provided by General Practitioners (GP) and specialists as well as prehospital emergency services and hospital care. In the capital region of Denmark there is one hospital trust, six University Hospitals and 40.000 health care employees. The CPH EMS works together with 29 municipalities, four police regions and seven fire and rescue services [27]. The CPH EMS ensures free, equal and 24/7 access to EMS [23, 44, 45]. The organizational structure of the CPH EMS is illustrated in Fig. 2.

The responsibilities for emergency preparedness and pre-hospital care planning are defined by the national Executive Order on Planning of Emergency Preparedness:

*“§ 4. The purpose of the pre-hospital effort is to save lives, improve health prospects, reduce pain and other symptoms, shorten the overall course of the disease, provide care and create security.” (translated via Google Translate) *[44]

### Data collection

Qualitative data was collected from April to June 2021 through a scoping literature review [47], and expert interviews.

#### Scoping review

A scoping literature review was conducted in May 2021. PubMed, Google Scholar were searched and supplemented by web-based Google searches, snowball sampling for grey literature, contacting professionals in this field, and reference tracking. The obtained literature was organized using the reference manager Mendeley© and screened for eligibility in three stages: title, abstract, and full-text screening. Details on the search strategy including search terms, and eligibility criteria are provided in Additional file 1.

#### Expert interviews

Twenty experts on CPH EMS were identified through recommendations and authorship of relevant publications and invited via email (cf. Additional file 2). For each WHO ECSF Matrix Domain, at least two experts were assigned with some covering multiple domains. To ensure completeness, the list of potential interviewees was independently verified by a senior-level executive and a senior-level researcher of the CPH EMS.

Individual interviews were conducted in person or via MSTeams, and lasted about 30 min each. Semi-structured, member-checked and pilot-tested questionnaires guided the interviews, and all but one conducted during ongoing operations, were audiotaped and transcribed non-verbatim by SB. The Comparative Method for Themes Saturation (CoMeTS) determined the number of interviews, continuing until no new information emerged or no further experts were available in the field of interest within the timeframe [48]. Each interviewee was asked to suggest specific topics and best practice components of the CPH EMS, and since no further recommendations were made, thematic saturation was assumed. No repeat interviews were conducted; clarifications were made in person or via e-mail. Transcripts were returned to the interviewees for verification or comments, with one clarifying comment provided for the non-audiotaped interview.

The scoping review and interviews were conducted concurrently, enabling an iterative process where literature findings informed more detailed interview questions, while expert insights helped contextualize study results—particularly when interviewees were also study co-authors [49].

Based on The Merriam-Webster Dictionary definition of ‘best practice’—“a procedure that has been shown by research and experience to produce optimal results and that is established or proposed as a standard suitable for wide-spread adoption” [50]—system components were considered as relevant if they met either of two criteria: (i) research-validated improvements to the status quo practices published in peer-reviewed journals (ii) practices or components deemed ‘good or best practices’ by the interviewed experts.

### Data analysis

A partial system analysis of the CPH EMS was conducted using directed (relational) content analysis [51], a method that builds upon prior research, using concepts or variables as initial coding categories.

Interview transcripts were coded deductively on pre-established themes derived from the WHO ECSF-Matrix [52] by two researchers (1st coder: SB, 2nd coder: TK) using Atlas.ti software, which aligns with the WHO Health System Building blocks: (i) human resources and training, (ii) essential medical products, technologies and infrastructure, (iii) information and research, (iv) financing (represented as separate core component in the WHO ECSF rather than a cross-cutting building block), and (v) leadership and governance. Document data were analyzed similarly, with translations provided by Deepl.com for non-English documents This coding approach accords with the first part of the prehospital EMS assessment tool by Mehmood et al. [37], evaluating inputs, capacity, and performance.

## Results

### Characteristics of sources of evidence

#### Scoping review

**Fig. 3:**
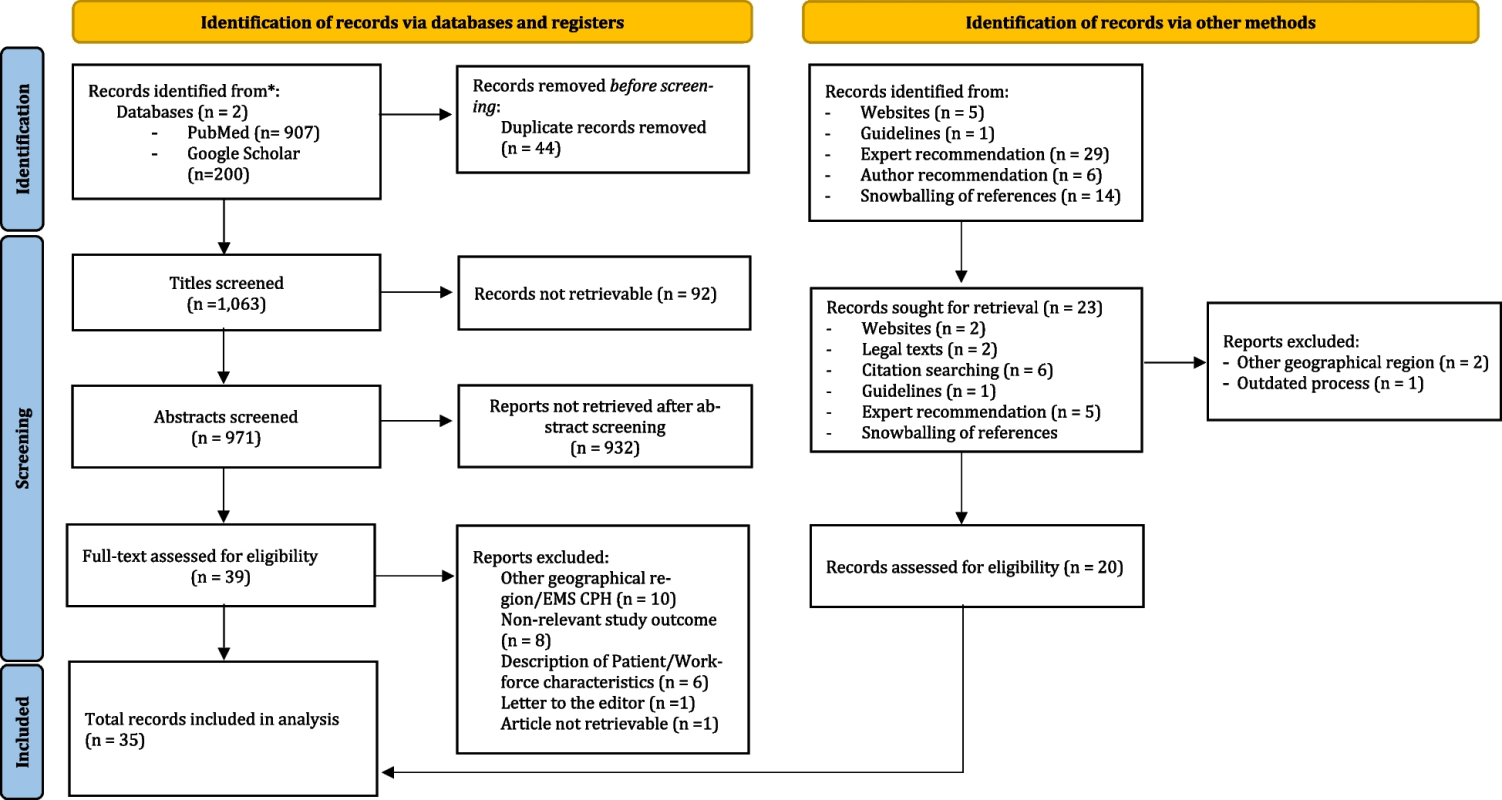
PRISMA 2020 flow diagram for new systematic reviews which included searches of databases, registers and other sources [53]

The literature selection process is depicted in the PRISMA 2009 flow diagram (Fig. 3). A total of 35 records were identified for document analysis. Of these, twenty-eight records were peer-reviewed journal articles, sourced through a database searches (*n* = 14), snowballing of references (*n* = 6); expert recommended literature (*n* = 5), and targeted unsystematic online searches (*n* = 3). Grey literature consisted of webpages/legal texts (*n* = 3), annual reports, and internal documents from CPH EMS (*n* = 4).

#### Expert interviews

From April through June 2021, we interviewed thirteen experts (referenced as E1 to E13). Four of the interviewees (30.8%) were part-time researchers and part-time employed within EMS as medical physicians (*n* = 3) or paramedics (*n* = 1). Seven interviewees (61.5%) were professionals currently working at the level of senior executive and/or operational management at the CPH EMS. One interviewee (7.7%) was a full-time researcher at the CPH EMS and one (7.7%) was employed at the operational level at a hospital in Copenhagen. Five invited individuals did not reply to the invitation and two individuals declined, stating a lack of expertise in the requested area. Experts covered at least one, and in some cases two domains of the WHO ECSF: Scene (*n* = 6), Transport (*n* = 3), Facility (*n* = 2), Cross-Cutting Elements (*n* = 6) with focus on financing, quality improvement, patient safety, research. Preparedness planning, service delivery, data management.

### Synthesis of results

The identified system components were charted in the four segments below that correspond to the WHO ECSF-Matrix’ categories: (i) scene, (ii) transport, (iii) facility, and (iv) cross-cutting elements (Tables 1, 2, 3 and 4). Data from the scoping review and interviews are synthesized under each topic as they address the same object of study and include overlapping perspectives, as some authors also participated as respondents.

**Table 1:** Identified components of the Copenhagen Emergency Medical Services on “scene”

*Site*	*Component*	*Aim*	*Detailed Description*	*References*
**SCENE**	**Mandatory BLS courses**	To enable volunteer laypersons as first aid responders and EMS system activation through bystanders	Education of the public in resuscitation: Mandatory resuscitation trainings in elementary schools (since 2005) and when acquiring a driver’s license (since 2006). The Copenhagen Tool is used to assess quality and adherence to European BLS guidelines of BLS educational activities nationwide	[24, 54, 55] [E3,E12]
	**National volunteer-based AED Network** linked to the EMCC (since 2006)	Dissemination and Registration of publicly accessible geo-coded AEDs	Publicly accessible AEDs are voluntarily purchased and registered by laypersons. Information on AED location and accessibility is publicly accessible via a webpage (www.hjertestarter.dk) and a smartphone-application (app) and is further linked to the emergency medical dispatch centers who follow nationwide standardized protocols for dispatcher-guided assistance of CPR and nearby AED retrieval in case of suspected cardiac arrest. Stored data from applied AEDs (e.g. ECGs) can be systematically retrieved and analyzed for research (e.g. to guide public education; post-resuscitation treatment and care).	[24, 56–60] [E1,E3,E8,E11]
	**“Heart Runner” Project**	App-dispatched citizens in case of suspected OOH cardiac arrest	A layperson above 18 years of age, that signed up for the “Heartrunner” app, may be dispatched by the EMCC as citizen responder. The citizen responder system alerts 20 citizen responders in case of suspected cardiac arrest and instructs them to retrieve the nearest AED as indicated in the app (using accessibility information form the AED network) or immediately visit the patient to perform CPR and BLS. Protection measures include an electronic survey after each event, assessing the performed activities, physical injury or danger (e.g. when crossing a busy street) and offers psychological support. In defined cases no citizens will be dispatched (e.g. when involving children < 8 years, in case of suicide attempt or in “unsafe surroundings”). Citizen responders may also decline to be dispatched. The Heart Runner Project was piloted at the CPH EMS before expanding nationwide.	[56, 61] [E1,E3,E8,E11]
	**On-site multidisciplinary collaboration**	To coordinate between services of healthcare, public health and public safety	In case of emergency medical situations, the **Joint Rescue Coordination Center** (part pf the Danish Defense) may be called to assist and provide search and rescue services. Healthcare professionals follow a three-week “joint incident command training” with police and fire brigade commanders.	[23] [E2]
			**The Tactical Emergency Medical Services (TEMS)** is a multi-sector collaboration, where the police accompanies the physician to reach the patient in tense situations where the health emergency response would normally wait until the police had secured that areas.	[42] [E6]
	**Emergency number (112) and medical helpline (1813)**	Single point of entry to all EMS services To provide easy access and relieve pressure on EDs and possibly on GPs immediate workload	**The Danish emergency number 112** covers all urgent emergencies, incl. police, fire department and EMS. All 112 calls are answered by the police, and in parts by the fire brigade. In case of a medical emergency, the call is forwarded to one of five regional EMCCs.	[23, 24, 42, 62] [E3,E8,E11]
			**The Medical Helpline 1813** of the Capital Region of Denmark is operated 24/7 by specially trained nurses and paramedics as call-takers who are supervised by physicians that may provide: • medical telephone self-advice, • referral to GP, • home visits (performed by the physicians from 1813), • face-to-face consultations by a physician employed at a hospital, • ambulance care (or one of the specialized mobile units), or • referral to a hospital or psychiatric institution for consultation or admission • On-site medical direction (chief emergency physician) is available 24/7 Both numbers are free and accessible 24/7/365 from all phones. Daily surveys via SMS are sent to patient callers to measure patient satisfaction.	[23–25, 45] [E4,E5,E9,E10,E13]
**DISPATCH**	**Emergency Access Button** (EAB)	To facilitate immediate access to medical help	Allows 1813-callers to bypass the telephone waiting line if the caller requires immediate medical action. In one study approx. 3% of the callers perceived their need to be of great urgency and used the EAB to bypass the waiting line, The EAB further seems to increase patient satisfaction with the OOH services and perceived patient safety in callers.	[63–67]
	**Medical and Technical Dispatchers** (since 2011)	Pre-assessment and adequate dispatch response according to the identified need	Healthcare professionals (registered nurses, paramedics and supervising physicians with various medical specialties or in specialty training) operate as dispatchers in the EMCC. **Medical dispatchers** can • support laypersons in the recognition and first response to life-threatening symptoms, • provide medical advice to the patient and • send the right response based on the assessed symptoms The dispatcher may respond with several actions as described above “Emergency number (112) and Medical Helpline (1813). **Technical dispatchers** are responsible for the logistics of simultaneously dispatching the appropriate emergency care vehicles corresponding to the level of urgency as assessed by the medical dispatcher. After the vehicle is dispatched, the medical dispatchers may forward further information on the patient to the ambulance personnel (as digital notes).	[23, 24] [E4]
	**Advanced Mobile Location (AML)**	To rapidly and precisely locate 112 callers	The system transmits the exact location of the incoming mobile phone (Android and iOS) to the EMCC. Still, the dispatcher will also ask the caller for the location.	[E2]
	**Video-transmission of scene to the dispatcher** 112 and 1813	To provide the dispatcher with visual information	Closed-circuit television (CCTV) helps to improve the dispatchers understanding at the scene (incl. information on patient and bystander’s response and physical environment), the communication with bystanders and their ability to guide the bystander (e.g. in performing correct CPR in case of a cardiac arrest). In 2020, video-visitation became an integral part of 1813 on scene to enhance timely understanding and adequate dispatch response.	[24, 68, 69] [E1,E4]
	**Call-back services** 112 and 1813	To reassure patient and ensure that condition does not deteriorate	In 112 calls and category “B” ambulance runs, the medical dispatchers calls the patients if the ambulance has not arrived within 25 min to follow up on the patient’s condition and adapt the emergency response if necessary.	[E4]
		To ensure that children well-being and increase safety	The 1813 dispatchers call back families with sick children staying at home to follow up on the child’s condition and ensure that they really do not have to be admitted to the hospital. This further provides immediate feedback and learnings to the medical dispatcher.	
	**Artificial Intelligence (AI) Speech Recognition**	To support dispatchers in identifying OHCAs (quicker)	Machine learning to recognize OHCAs through a speech recognition software to support the dispatcher in sending the right response quicker. As a pilot study suggests, the AI software appears to be more accurate and faster than medical dispatchers taking the call. For this, the Digitization Award within Innovation was assigned to the CPH EMS in 2020.	[23, 70, 71] [E2,E4,E11]
	**Criteria-based blue light dispatch**	To determine the level of urgency of the medical emergency	**The Danish Index for Emergency Care** is a criteria-based dispatch decision support tool that aids to determine the most adequate dispatching response (since 2011). The Danish Index is a computer-aided click-system of 37 main criteria (or 38 if category “unclear” is included) that correspond to clinical signs, symptoms or incidents. Based on this, the medical dispatcher assesses the level of urgency (rather than diagnosing) and decides on the needed response (classified A through F; A = acute, potentially life-threatening condition; F = non-urgent).	[23, 72, 73] [E4]
	**Cross-sector collaboration** Between EMCC, EDs, GPs and municipalities	To benefit and improve patient care	Collaborations between 1813, emergency admissions and clinics discuss and agree on initiatives to improve patient care. Strengthened cooperation with GPs include the management of deceased persons and the training of medical doctors. Collaborations with patient associations ensure learning and adequate care and advice through medical dispatchers at 1813.	[23, 24]
	**Needs-based dispatching of vehicles**	Combinable system response options	Dispatching of multiple ambulance vehicle and staff based on geographical location, chief complaint and number of patients (e.g. mass casualty incident). Example: In case of cardiac arrests physician-manned mobile emergency care units (MECUs) will be dispatched alongside the ambulance providing ALS (incl. the use of defibrillators), sometimes referred to as rendevouz.	[23, 24, 42, 43]
		Acute and non-acute responses	The EMCC further manages non-acute ambulance runs and transports including scheduled ambulance tasks and interhospital transfers. Hospital wards, GPs and others may contact the EMCC for both acute and non-acute transportations.	[23–25] [E2,E5]
	**Integration of GPs and specialists**	Gatekeeper to EMS Cooperation with and referrals to in-hour healthcare services	GPs may refer patients to other specialists, EDs or specific hospital wards and can request ambulances by calling the EMCC. GPs perform home visits and/or consultations via the EMCC. Close collaborations between 1813 and healthcare specialties such as the eye department, gynaecology, rheumatology, pediatrics and cardiology, as well interdisciplinary initiatives such as stroke to strengthen referrals.	[23, 25]

**Table 2:** Identified components of the Copenhagen Emergency Medical Services during “patient transport and care”

*Component*	*Aim*		*Detailed Description*	*References*
**Needs-based transport care** Supplementing specialized mobile care units	**Mobile Emergency Care Unit (MECU)** Physician-manned vehicle		Emergency cars, staffed by an emergency physician and paramedic that may provide advanced medical diagnostics and treatment, providing support to the region's ambulance services	[24, 42, 43, 74] [E3,E4,E6,E11]
	**“Sociolancen” or Mobile Health-/Social Care Unit** *For “socially deprived” citizens*		The “Sociolancen” provides acute health-related and social-related care and is staffed with a paramedic from the CPH EMS and a social healthcare worker from the Social Administration of the Copenhagen Municipality and is stated to be “bridge-building between social and mental services”. It is a collaboration between the Capital Region, the City of Copenhagen and the Capital Emergency Management Agency,	[24, 42, 75]
			Transfers occur to somatic and psychiatric EDs or shelters while some patients are left to self-care. Referrals may also be made e.g. to police or emergency ambulances.	
	**Mobile psychiatric critical care unit (MPCCU)** *For psychiatric emergency cases*		The MPCCU is a special and spacious vehicle with a “blue-light response option” and may be escorted by the police. It is manned by an on-duty paramedic and an experienced psychiatrist from Regional Mental Health Services. The psychiatrists may be consulted in case of a mental health emergency, are on-call during OOH times (GPs are responsible during office hours) and are picked up by the paramedic on duty. The psychiatrist provides in-depth knowledge of regional and community mental health services, has access to relevant electronic patient records.	[24, 42] [E9]
			Approx. 60–70% of calls to the psychiatric emergency service are handled via telephone consultation or referral. In the remaining 30–40% of the cases, the psychiatrists have direct contact to the patients.	
			The MPCCU may be dispatched by the EMCC (112 or 1813), the police, social welfare services, ambulances or somatic mobile critical care units (MECUs).	
**Needs-based transport care (II)** Supplementing specialized mobile care units	**“Babylancen”** or Mobile Neonatal Intensive Care Unit		Manned by on-duty paramedic around the clock and staffed by neonatal team from University Hospital Copenhagen. Ability to transport parents and provides a video feed above the child.	[24, 42, 76]
			It is designed to transport premature or/and critically ill children to a specialized hospital with an intensive care unit (“Rigshospitalet”).	
	**Prehospital Visitation (PHV)** *For lower acuity cases*		A medically fully equipped vehicle manned by one paramedic for scheduled “B” runs (non-time critical), offering personal contact to the patient to find the best solution of care. The PHV further creates a greater link between 112, the ambulance staff and municipal emergency teams.	[24, 42]
	**National Helicopter EMS** *For short response times*		Four helicopters provide 24/7 national coverage for short response times in urgent situations or remote areas. Each is staffed with a pilot, an anesthesiologist, and a specially trained paramedic and may transport one patient.	[23, 24, 77, 78] [E6]
	**Mobile Causality Clearing Station** *For large events*		Emergency trucks staffed with paramedics and hospital nurses in case of a major incident with many causalities. It is staffed by paramedics and ambulance personnel and can hold up to 40 patients at once. Further use cases include large planned events (e.g., marathons or festivals) and serving as temporary hospital facilities, such as during a hospital reconstruction in 2019.	[24, 42] [E8, E9]
	**Multi-tier system**		Three-tier response system:	[23, 42]
	*Multi-level responses with regards to mobile care unit and staff*		1. Basic level: ambulances (Basic Life Support) 2. Intermediate level: paramedic- or nurse-manned cars 3. Advanced level: prehospital physician (consultant anesthesiologist) in cars or helicopter (Advanced Life Support)	[E3,E5,E6]
	**Rendezvous Model** *Tailored dispatch responses e.g. transport or immediate care on-site*		MECUs (see above) may be sent to meet the ambulance on scene or during transport. The aim is to support the ambulances’ BLS care as the ambulances are staffed with emergency technicians (EMTs) and/or paramedics.	
	**“Sygetransport”** *Non-emergent / low acuity transport-*		Alternative form of laying or seated patient transport, for patients that are not acutely ill but still need to go to the hospital. This avoids unnecessarily binding ambulance resources while providing needs-based care.	
	**Medical Delegation for ambulance staff**		Ambulance personnel are authorized as healthcare professionals (since 2019). The regional prehospital medical doctor has the overall responsibility for the regional prehospital treatment. There are several opportunities to diagnose and/or treat patients inside the ambulances.	[23, 43, 44] [E11]
**Digitized Documentation**	**Ambulance Diagnosis** **/treatment** *For early and adequate treatment*		**Telemedical Support & Point-of-care technologies** For early recognition, timely and correct treatment or transport to the correct treatment site e.g. when stroke or coronary infarction is suspected. Medical direction of the ambulance personnel by specialized units e.g. thrombolysis centers or coronary intervention centers. Point of care technologies allow for early (preliminary) diagnosis and treatment on-site or inside the ambulance.	[24, 43] [E2, E6, E11]
	**Pre-hospital Electronic Patient Care Record and Registry** *Prospective patient registration of every transported patient that is accessible by the hospital personnel*		All EMS providers nationwide use the same electronic prehospital patient record system (“Præhospitalet Patient Journal”, PPJ). Ambulance personnel enter patient information on all prehospital contacts of the patient with the EMS on a specially designed tablet. Patient information include: prehospital findings, examinations and treatments and the hospital the patient is admitted to. A physician on scene may assign a diagnose to the patient. ICD-10 diagnoses are available in the PPJ. Dispatch and hospital ambulance can access the PPJ. The national PPJ forms the basis for the Danish Database for EMS.	[23, 28, 43] [E6, E13]
	**Wireless Sensors Network**		Measured patient data (e.g. Electrocardiograph (ECG), blood pressure) are being transferred to a computer, transmitting real-life vital data to the PPJ, to monitor and document the patient’s vital parameters during transportation. The hospital may access this data and the ECG can be transferred to a specialized cardiology center.	[E6, E13]
**Data Management & Development**	**EMS Research Center**	To develop and improve service quality regarding patient contact and treatment at the CPH EMS	Research in the EMS is highly prioritized, and implementation ways from research to practice are short. The research projects emerge from actual problems and relevant clinical cases.	[23, 24, 46] [E1, E8, E11]
	**Public–Private Partnerships**	Development of smart technology	Computer-aided dispatch decision support tool (Danish Index for Emergency Care) translates caller information in recommended pre-hospital response. The AI speech-recognition-software that recognizes OHCAs was created by a private company Corti (Corti.ai, Denmark).	[23, 24, 43, 70] [E10]
		Research Funding; Sponsoring of Technology and Public Campaigns	Private foundations like TrygFonden or Lærdal foundation provides funding for research projects.	[23, 44] [E1, E2]
	**Danish Quality Data- base for Prehospital EMS (QEMS)**	To establish national quality assessment and improvement through data monitoring of the PEMS	Data included are information on the PEMS (calls, ambulances and patients) which may be linked to the in-hospital medical records and registries and allow to follow the “EMS patient care pathway” including access to clinical data and follow-up after PEMS care.	[23, 24, 29] [E7, E10]
			Monthly (aggregated data at a regional level) and annual (aggregated open-source data at a national level) reports provide incentives for quality improvement.	
	**Sharing Data for Care Optimization**	For optimizing care, planning and data driven management and research	Electronic pre-hospital charts (“PPJ”), EMCC Database, referrals and “discharge” notes at the CPH EMS. Linkage with nationwide Danish registers is possible through the unique personal registration number.	[24] [E2, E10, E13]
	**Integrated ICT System**	To communicate throughout all units of the EMS and aggregate data for monitoring, planning, quality improvement and research	Integrated communication across all EMS units and the hospital system. Notes may also be referred to the ED and GP. The integrated system produces a vast amount of prehospital data which is being summarized in reports to the executive level of the EMS and used for planning, research and quality improvement.	[24] [E7, E10, E13]
**Service Delivery**	**Patient Safety & Satisfaction**	Danish Patient Safety Data- base Systematic and citizen-oriented complaint handling	Mandatory reporting to the database and data analysis of the core of the problem (forwarded to hospital if it is also concerning them) to ensure patient safety. In case of complaint, the citizen is being called two days after forwarding the complaint to resolve or clarify the complaint.	[79–81] [E7]
		Patient involvement	The patient perspective is included in external audits of 1813. Citizen workshops for feedback on EMS-related functions such as 1813 and “Sygetransport”	[43, 65]
			Weekly surveys to measure citizen satisfaction related to experiences at the EMCC and patient transports through a collaboration with KOPA (Competence Center for Patient Experiences)	
	**Integrated Education**	University Course	A university course in prehospital emergency medicine, including internships at EMS, has been offered since 2020.	[43] [E9]
	**Integration of Health Sectors**	Psychiatric Emergency Services	The Psychiatric Emergency Preparedness of the Capital region of Denmark is a collaboration between the Emergency Prepared- ness (EMS) and the Psychiatry of the Capital Region of Denmark. The unit is staffed by a psychiatric specialist and medical assistant. Such a specialized psychiatric emergency service exists only in few European regions.	[24, 43] [E5]

**Table 3:** Identified components of the Copenhagen Emergency Medical Services in the inpatient “facility”

*Area*	*Component*	*Aim*	*Detailed Description*	*Ref*
**Emergency Department**	**Gatekeeping system**	To be prepared for the patient’s arrival and avoid long waiting times and overcrowding of EDs	Mostly, patients need to be referred to the ED by a GP or a nurse. It is allowed for them to enter the ED on their own account, but it is not favored.	[23, 24, 62] [E13]
	**“Copenhagen Triage Algorithm”**	To prioritize most urgent emergencies (Emergency Process Triage)	Five color-coded triage system categorizes patients according to the level of urgency from red (resuscitation, re-evaluation every 0 min.) to blue (minor injuries or complaints, re-evaluation every 240 min.) based on vital signs and clinical assessments by ED nurses.	[82] [E5, E13]
**Central hospital data-**	**Acute Admission Database** (Hillerød Hospital)	To create a database to easily analyze potential associations between patient status at admission and several outcome measures	Collecting data on patient status of admission (incl. vital signs, complaint, and triage category), blood sample results and outcome measures (incl. length of stay, in-hospital mortality and mortality seven and 28 days after admission, discharge diagnosis).	[83, 84]

**Table 4:** Identified components of the Copenhagen Emergency Medical Services as “cross-cutting elements”

*Area*	*Component*	*Aim*	*Detailed Description*	*References*
**Research & Information**	**Nationwide registries,** **e.g. Danish Cardiac Arrest Registry, National Patient Registry**	Possibility of population-based research and quality improvement for the complete patient pathway	Research is a priority of the CPH EMS. Over the past decade, the research focus has shifted from hospital-based diagnostics and treatment to also include pre-hospital settings. The prehospital registries are being supplemented by other healthcare registries, covering patient contacts with hospitals, in-hospital diagnoses and treatments as well as population-based information (e.g. education, living conditions and wealth), and total consumption of physician-prescribed drugs in Denmark	[23, 24, 28, 60, 72] [E11]
			Linkage of different registries with the unique civil personal registration number enables monitoring of the whole patient pathway from first contact of the EMS through the prehospital treatment, diagnostic patterns, and outcomes (e.g. mortality or return to work) and follow up.	
	**International Cooperation and Knowledge Exchange**	European EMS Leadership Network & Global Resuscitation Alliance (GRA)	The network consists of nine European EMS systems working together to create a coherent European EMS system. The network collaborates on research and shares knowledge and best practices. The GRA consist of four international EMS organizations working together and exchanging knowledge to increase patient survival after cardiac arrest.	[23, 24, 43]

(i) Scene

The EMS response begins the moment someone recognizes a need for medical help. As one expert put it,

“*as soon as someone realizes that they need an ambulance, or they need help and they call 112 or 1813. The taking care of the patient starts there.” (E2).*

Citizen involvement is encouraged through regular mandatory Basic Life Support (BLS) courses [54, 55] (E12), and citizen responder programs such as the “HeartRunner”-Project, where app-dispatched citizen can provide resuscitation support [56] (E1, E3, E11), which relies on a national network of accessible Automated External Defibrillators (AEDs) largely procured and funded by the Danish people or institutions [56–61] (E1, E3).

The Emergency Medical Communication Center (EMCC) serves as a single point of access by integrating the emergency number 112 and medical helpline 1813 and using the same integrated IT-system [24]. The EMCC is available 24/7/365 and staffed with medical calltakers (nurses and paramedics) and technical dispatchers. One expert labels this integration of both numbers as the most important innovation:

*“the most important innovation in my opinion being implemented in Copenhagen since *[20]*14, is that you just make a decision whether to call 1813 or 112 and then, what’s happening behind in the black box is going to be decided by us [the EMS personnel]. […]. An integrated patient care system that can provide help 24/7 independent of what is your need and what time of the day.” (E8)*

The triage process is supported by computer-based protocols (i.e. “Danish Index”) [72, 73] and may be supported by an artificial intelligence-based (AI) speech-recognition software in case of suspected cardiac arrest. In a recent trial, the AI demonstrated a remarkable sensitivity, accurately detecting 85% of cardiac arrest cases over the phone, showing a significantly higher sensitivity than human medical dispatchers [70], highlighting the potential of AI to enhance early recognition and response in critical situations. Communication with the patient or bystander can be extended through video-transmission and video-aided telephone CPR [68, 69] (E4). In case of an emergency, the waiting line of the non-emergency medical helpline 1813 may be cut using an Emergency Access Button (EAB) [63–67].

The dispatching of prehospital resources may include different multilevel responses and is best matched to the patient´s needs [62]. GPs are integrated into the EMS system by supervising and supporting calltaking and dispatching activities during OOH, conducting home visits, and may order ambulance transports or book ED appointments via the EMCC. Referral or discharge notes are automatically extracted from the patient charter and forwarded to the relevant recipient, ensuring seamless information transfer between healthcare departments [24].

Upon arrival at the scene, prehospital personnel can initiate treatment and, in some cases, perform diagnostic assessments, enabling timely and needs-based care (E2, E6). In Denmark, paramedics can decide against hospital transport based on legal guidelines (Bekendtgørelse om ambulancer og uddannelse af ambulancepersonale. BEK nr 1264 af 09/11/2018). Patients who are not conveyed may receive on-scene treatment, be discharged without follow-up, or be redirected to primary care services [24]. All ambulance services are part of EMS and work under the same Standard Operating Procedures with medical supervision 24/7 [25].

(ii) Transport

A variety of specialized mobile care units supplement the traditional ambulance vehicles such as ambulances, physician-staffed mobile critical care units and helicopter EMS to provide patient-tailored and differentiated care. This includes “Sociolancen” for socially deprived persons [75, 85], “Babylancen” for young children and their parents [76], mobile critical care units [74], also for psychiatric cases [24], national helicopter EMS [77, 78] and mobile causality clearing stations for mass casualty incidents [24, 42, 43]. Units may be dispatched alone or in combination (“Rendezvous Model”) and manned with different level trained staff with varying (delegated) competences of treatment. Additionally, non-emergency transports are being coordinated by the CPH EMS, including “Sygetransport”, for laying or seated transports of non-critical patients to and from treatments [24, 43]. Technical solutions include telemedicine for medical support, wireless sensors network for automatic collection and documentation of vital signs, and a prehospital patient journal for information, communication and documentation of patient transport and care [23, 24] (E6).

One expert describes the growing possibilities within the prehospital EMS as follows:

*“if the patient needs to go to the hospital - there are several opportunities also now, not only to diagnose, but also to treat patients. So, moving from an ambulance just being a transport form to a hospital doing the diagnostic and the treatment, more and more things are now shifted towards the prehospital phase”. (E11).*

(iii) Facility

Although the CPH EMS is responsible for prehospital resources, the following examples were included as they illustrate the collaboration of pre-hospital and in-hospital care. This begins with the in-hospital environment which is largely triaged via the dispatchers at the EMCC or the GPs, and the “Copenhagen Triage Algorithm” that categorizes incoming patients [82–84] (E13). An early information transfer between ambulance is “[…] *a big improvement in the hospital, [and helps] being prepared of what ‘s the matter with this patient arriving to our emergency room within the next few minutes.*” (E3). The “Acute Admission Database” records the patient pathway to aggregate patient-based data for analysis and quality improvement [83, 84]. Due to the prehospital focus of the CPH EMS, limited data was available for the domain “facility”.

(iv) Cross-Cutting Elements

The optimal coordination and integration of cross-cutting elements of the CPH EMS was summarized by one expert as *“It takes a system to save a life”* (E8). *“Getting the right patient to the right treatment at the right time.”,* a declared goal of the CPH EMS system, is thought to lower redundant expenses while increasing care quality [24]*.*

Identified components regarding cross-cutting elements, predominantly address the collection, monitoring, and evaluation of information, supporting research and quality improvement. Data is used to monitor patient needs and systems performance, or as summarized by one expert: *“data save lives”* (E10).

Patient needs can be monitored through comprehensive data collection, linking EMS activity with individual-level health data via the Danish civil registration number [28], patient satisfaction surveys [63, 65, 86], project-related surveys [56, 69], and annual benchmarking of prehospital data [87], allowing the tracking of patient pathways, highlighting gaps, and supporting research-driven improvement. Patient safety is improved through tools like the national incident reporting system, the ´Danish Patient Safety Database’ allowing patients and professionals to report incidents, fostering a system-wide learning environment for improvement [29, 79], following the mantra “*improve the system– not the person*” [80, 81].

A variety of data is being collected through performance monitoring of the EMCC (including data on call and mission processing, waiting times, hospitalization rates, patient satisfaction and complaints, home visits by GPs etc.), registries and databases contribute to research and quality improvement, while regular data-based quality improvement councils (E7, E12), and annual public reports [88] ensure high transparency and accountability of the CPH EMS. An integrated ICT system incorporates the emergency dispatch of prehospital resources, enabling communication and data aggregation of the operational side of the CPH EMS*.* This highlights the data-driven approach of service delivery at the CPH EMS and its focus on research, facilitated by public–private partnerships for development and maintenance of research projects and innovations [24, 42].

## Discussion

**Fig. 4:**
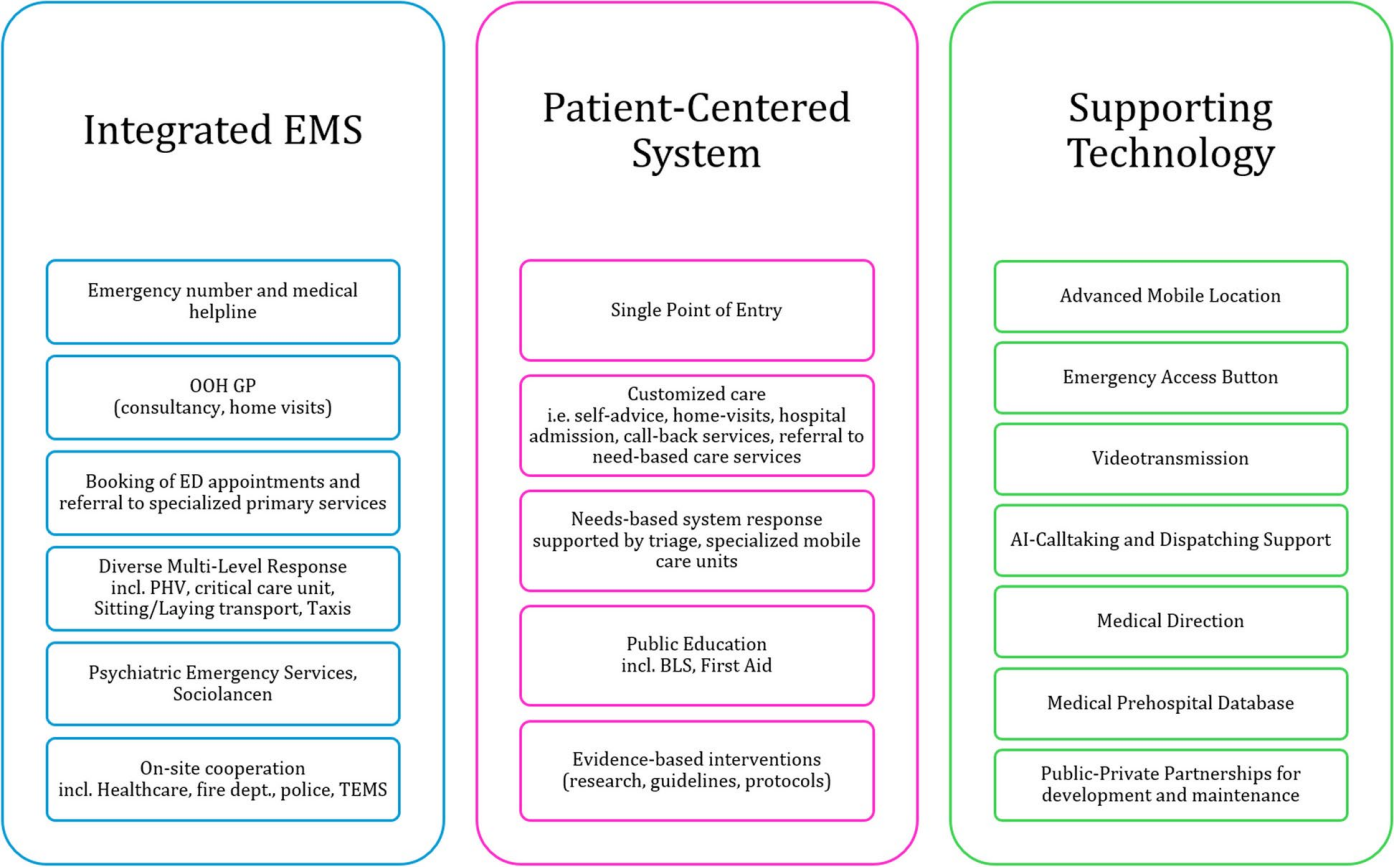
Summary of examples for integrated, and patient-centered components and supporting technologies at the Copenhagen Emergency Medical Services

**Fig. 5:**
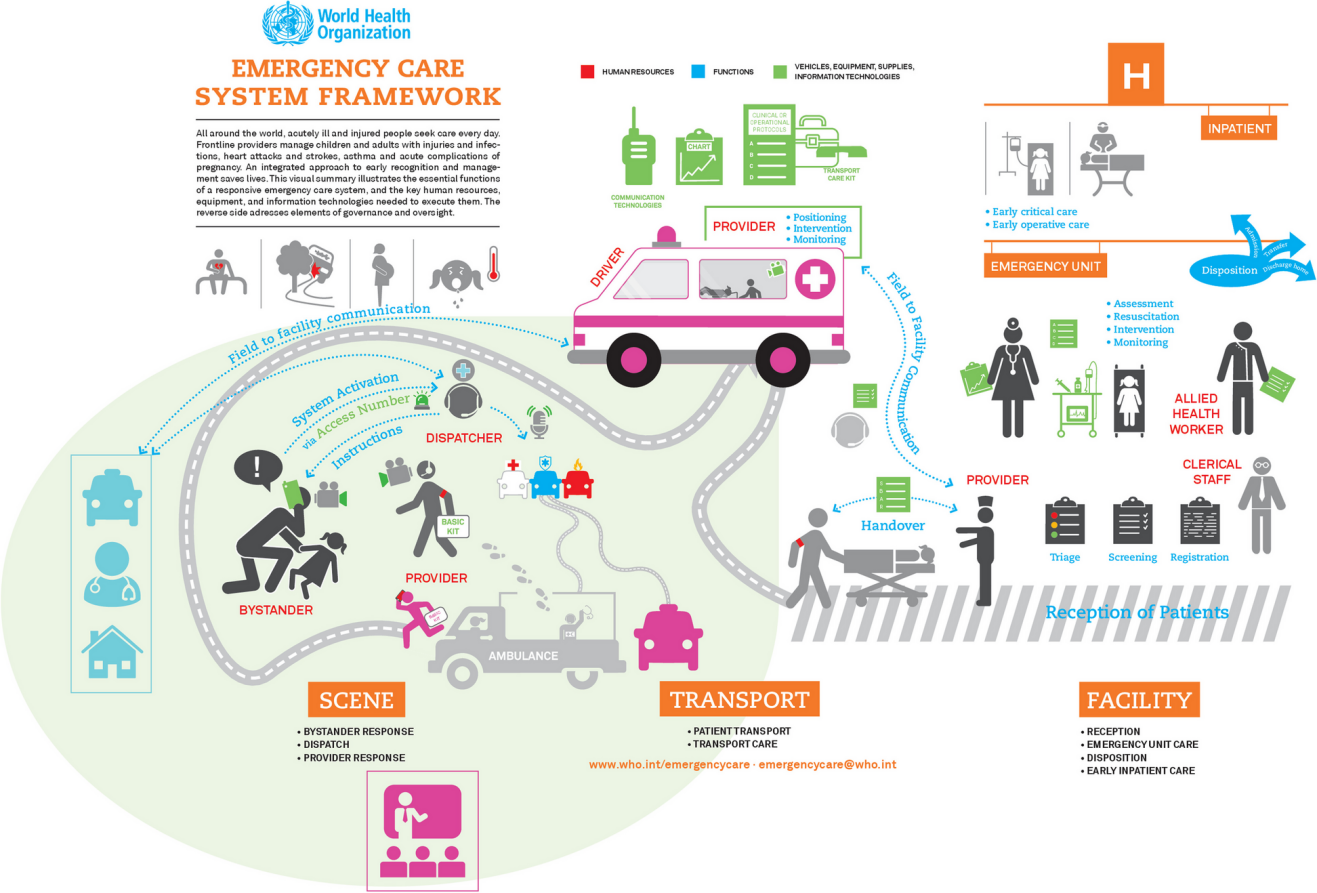
Amended WHO Emergency Care System Framework incorporating examples of evidence-based practices at Copenhagen EMS to demonstrate an integrative approach in emergency care (amended from [22] with kind permission of emergencycare@who.int obtained on 04.04.2023). Color-coded elements highlight key components: integrated EMS care within the context of primary healthcare (light blue), patient-centered EMS care (pink), and supporting (smart) technology (bright green). The rain-drop-shaped area highlighted in light green accentuates the focus on integrated and preventive prehospital emergency care provision

The qualitative assessment of the CPH EMS system highlights several examples of (i) integrated, and (ii) patient-centered emergency care, and supporting (iii)– sometimes “smart”—technology solutions (see Figs. 4 and 5). These findings clearly indicate a shift towards prehospital and community-based emergency care, moving away from a mere hospital-centric model.

The identified key components of the CPH EMS were validated through member checking with researchers at the CPH EMS and were subsequently mapped onto the WHO ECSF as follows:

Blue components represent examples of integrated EMS care, focusing on the triage process for differentiated responses, including dispatching of various specialized response units, self-help advice, and referrals to Emergency Departments (ED), GPs, specialists, or home consultations.

Pink components illustrate examples of patient-centered EMS care, including a “single-point of access” via the EMCC emergency line (112) and medical helpline (1813), app-dispatched responders, mandatory community BLS trainings, and needs-based on-site treatment or transport care by specialized EMS personnel and equipment.

Green components depict supporting– sometimes smart– technologies, such as urgent call prioritization, video-transmission, AI-based speech recognition for early detection of out-of-hospital cardiac arrest (OHCA), and telemedical supervision.

The mapped components of CPH EMS in the WHO ECSF infographic highlight the importance of community-based interventions and a needs-driven, differentiated system response (as shown in the raindrop-shaped light green area). This approach moves beyond traditional hospital-focused care, enabling on-site case resolution or redirection to the most appropriate care pathways, including EMS, primary care, and OOH services.

### Integrated EMS

The CPH EMS system exemplifies an integrated approach at different stations of the patient pathway within EMS.

Firstly, the EMCC serves as a ‘single point of contact’ for both emergency and non-emergency medical concerns (112 and 1813), optimizing patient navigation [23, 24, 27], early and systematic triage supporting optimal EMS resource allocation, effectively functioning as a gatekeeper, assigning unspecific cases to disease-specific care [52, 89]. Its aim to reduce ED congestion and improve EMS system performance is a priority shared by many healthcare systems worldwide [15, 19].

Secondly, CPH EMS offers a differentiated response through specialized units that address different levels of urgency and cover a broad spectrum of health, including mental and social care aspects [24, 42]. This development can be found in many EMS systems, reflected in the establishment of single-response, multiprofessional, and/or tele-medical supported mobile units [90–98].

Thirdly, EMS personnel may triage patients on-scene, opting against hospital transport [99]. This reflects a shift in many EMS systems from emphasis on hospital transport to providing advanced prehospital care, and redirecting patients to the best point of (available) service or discharging the patient without follow-up [100, 101].

The WHO ECSF could further emphasize an integrated approach, including a single point of access, comprehensive triage, and a differentiated system response and include care options of social, psychological, and sub-acute cases frequently managed by EMS, as well as EMS integration within the broader healthcare system. Aligning with the European Union’s ‘State of Health’ reports from 2017 and 2019 [102, 103], a stronger emphasis on integrated care systems could support policymakers and health system administrators in develop a holistic health system including the interaction of sub-systems.

However, transforming healthcare silos into an integrated system comes with challenges. During the CPH EMS restructuring, obstacles included “traditional thinking in hospital structure”, “facilities and logistics”, “stakeholder power (physician vs nurses, GPs vs other physicians)”, and “money” [27]. Similar to reports from Brazil integration efforts encountered policy, structural and organizational barriers despite improvements in care quality and health systems effectiveness [104]. Overcoming these challenges require sustained policy and organizational support to fully realize the benefits of integrated systems.

### Patient-centered emergency care

CPH EMS takes a patient-centered approach, addressing somatic, social, and psychiatric needs through differentiated care pathways, supported by a single-point-of-access, telemedicine, specialized mobile units, scheduled ED visits or primary care services [24, 27, 42]. Research from Australia has shown that one in ten EMS-attended patients presented with mental health issues, with most (74,4%) being transported to hospitals, despite being more suited for community-based mental health services [105]. This stresses the need for a holistic assessment, considering somatic, mental, and social aspects, while taking into account patient-specific settings and life circumstances.

In contrast, the WHO ECSF illustrates patients as individuals with specific emergencies—such as cardiac arrest, car accident, pregnancy, or pediatric illness— seemingly focusing on somatic emergencies with a singular response option: transport to a hospital. While this may not be intentional, it visually underrepresents the reality of complex patients’ abilities and needs.

Patient and bystander abilities include health literacy and health system literacy among further circumstantial factors, essential for recognizing type and urgency of medical needs and seeking appropriate care [21, 106]. While Copenhagen research projects observed a community with strong health awareness [66], and active support of initiatives such as the heartrunner project [56, 63, 64], the importance of early recognition and help-seeking behavior becomes even more apparent when reviewing examples with less favorable conditions. In a population-based survey in The Gambia, limited community awareness of common warning signs, especially for non-communicable diseases like stroke, acute coronary symptoms, or diabetic emergencies, was associated with a high proportion of disability especially among the young male population [107].

These reflections on patients abilities, needs and circumstances, have been emphasized by EU and WHO initiatives on integrated, patient-centered healthcare systems that advocate for “services of better quality, financially more sustainable and *more responsive to personal preferences and needs*”. The WHO Integrated and People-Centered Health Services Framework (IPCHS) states that “all people have equal access to quality health services that are *co-produced in a way that meets their life course needs*, are coordinated across the continuum of care, and are comprehensive, safe, effective, timely, efficient and acceptable; and all carers are motivated, skilled and operate in a supportive environment” [108].

### Supporting technologies in EMS systems

CPH EMS has integrated various supporting and smart technologies and have indicated in various studies that these may (i) improve timely EMS access, triaging, and preliminary diagnosis [24, 68, 70–72, 109–111], and (ii) facilitate seamless communication and documentation (E6) [24], enabling data transfer and communication across providers including GPs or inpatient care facilities.

Technology can support “seamless interaction” among care providers across settings and sectors as demanded by the WHO Framework on Integrated People-Centered Health Services [108], and enable tele-health and telemedicine care that can play a key role in reducing care disparities, enhancing health literacy, promoting healthy behaviors [112], and improve EMS efficiency [113, 114]. However, availability and quality of data input remains essential for effective care delivery [115].

Beyond operational benefits, technology facilitates digitized and standardized data collection, essential for monitoring, quality improvement, and allocation optimization. The role of standardized data in enhancing emergency care quality has also been emphasized by the 72nd World Health Assembly [116]. Similarly, Mowafi et al. stress the need for data-driven research to build evidence-based EMS services, noting a lack of emergency care surveillance and registries in most low- and middle-income countries, which could significantly improve service quality and Public Health [117].

The WHO ECSF aims to depict the “essential functions” of an EMS system. Thus, it is comprehensible that few digital technologies are included as these depend on resource availability and (digital) infrastructure. Nonetheless, smart information technology and modern biotechnology are believed to offer significant benefits by enhancing efficiency, accessibility and personalization in healthcare [118], a shift accelerated during the Covid-19 pandemic when digital care expanded rapidly [119]. Thus, a balanced approach—acknowledging the benefits of supporting technology while considering infrastructure limitations– could make the WHO ECSF more adaptable while including in which areas technological support could be beneficial if available.

While promising, smart technology in EMS also introduces risks, such as fallibility e.g. false-negatives in AI speech-recognition, or challenges in infrastructure and cybersecurity. Thus, ongoing improvements in IT infrastructure, skills, security, and data protection standards are essential.

### Transferability

EMS systems and patients’ needs vary widely due to differences in socio-demographics, culture, healthcare infrastructure, political and environmental contexts, and overall resource availability. While the WHO ECSF is a valuable framework, its strong focus on hospital-centered EMS limits its applicability, particularly in rural and low-resource areas where integrated community-based and telehealth solutions may be of even greater importance than in a hospital-dense area.

Similarly, CPH EMS components´ effectiveness is context-dependent, relying on a high-resource health system with fiscal stability, an effective and reliable approach for standards, guidance, and system organization; supporting a robust infrastructure, resources for specialized mobile units, and comprehensive data collection and linkage. While initiatives such as the community responder program succeed also due to cultural factors like widespread public first-aid training.

However, while CPH EMS offers examples of good practice, challenges remain, such as AED access in rural areas, data flow between prehospital and hospital systems, and integration with social and mental health systems– showing the need for continuous evaluation and further development. Nonetheless, the presented examples are largely backed by peer-reviewed research and practitioners working in CPH EMS, thus, confirming its evidence base and practicality in the CPH EMS setting, making them valuable considerations for the WHO ECSF.

Finally, it is important to recognize that good or best practices are generally ‘fluid concepts’ [50], meaning they constantly evolve with changing needs and new organizational and technological developments. Our considerations on how the WHO ECFS could be strengthened, is not intended to represent a final blueprint for the ideal EMS system but highlights areas for improvement and emphasize the need to for continuous review and updating of the WHO ECSF, to ensure that it remains a relevant and evidence-informed guide for EMS systems.

### Limitations

This study has several potential limitations. First, its qualitative and regional approach may introduce biases in data collection, including selection bias, researcher bias, and interviewee bias in expert interviews. Additionally, the geographical focus of the scoping review on the CPH EMS and, in some cases, the Danish perspective on nationwide structures, may have limited insights from other EMS models, and a broader literature review could have yielded additional findings for the WHO. To minimize the interviewee biases, expert selection was verified, and multiple data collection (interviews and literature) were to cross-check. Transcript verification and member-checking were also used to reduce interpretation bias. Publication bias was addressed by including grey literature and internal documents. However, no quality analysis of the included studies was performed. Second, due to resource constraints (time and personnel), only a partial system analysis was feasible, potentially leading to an incomplete representation of the CPH EMS system. Future research using a multifaceted approach, such as direct inspection/observation and focus group discussions, as suggested by Mehmood et al. (2018) could enhance system assessment. Third, as two sections of the WHO ECSF were not publicly available at the time of the study (E-Mail responses of emergencycare@who.int on 31 May 2021 and 04.04.2023), they were not considered. Lastly, data collection were conducted by a single researcher (SB), however, results were peer-reviewed and verified by senior-level researchers at the CPH EMS and Maastricht University to mitigate bias. Finally, given that this study focuses solely on CPH EMS, the components identified are not claimed as being unique or superior, thus are not considered ‘best practices’ or ‘unique to the CPH EMS’ as there might be other EMS systems with similar or even better practices and components that may be worth exploring in a different study.

### Implications for practice and research

The findings highlight the need for research-driven EMS systems to continuously measure, review and enhance current EMS practices and frameworks. The CPH EMS exemplifies the benefits of a close research-practice link, encouraging systematic data collection, rapid implementation of research insights, and fostering a culture of innovation. With short implementation ways from research to practice, making the CPH EMS an innovative and adapting system.

The WHO ECSF offers guidance for effective design of a patient pathway within EMS systems, but could be further developed in three areas: (i) integrating EMS within primary healthcare, public safety, and public health frameworks for a holistic approach, (ii) emphasize the EMCC´s role as the central point of contact and needs-based resource allocation and (iii) balancing its hospital-centered, resource-intensive model with guidance suited for low-resource or underserved settings.

As this study predominantly focused on the WHO Health System Building Blocks and the PEMS Framework from Mehmood et al. (2016), a subsequent step would be to further assess (ii) outputs (access, quality, coverage, and safety) and (iii) goals (improved health, responsiveness, social and financial risk protection, and efficiency) across the EMS, primary health and care, public safety and public health systems. Emerging stressors on the EMS and health system, such as cross-border care needs [120, 121] extreme weather events [122–124], the emergence of conflict areas (encompassing physical, political, and digital dimensions), have not been discussed within this paper but indisputably must gain greater significance in the design and resilience strengthening of health systems including emergency care systems.

## Conclusion

This study highlights components of an integrated, patient-centered and technology-supported EMS system in the Capital Region of Denmark, including (i) integration of EMS within public health and primary care, (ii) patient-centered strategies such as a single point of access, effective triaging systems, and diverse care response options, and (iii) the use of supportive technologies to enhance care coordination, operational efficiency, and patient outcomes. These findings advocate for incorporating evidence-based practices from a research-driven, integrated EMS system into the WHO ECSF, emphasizing a shift from a hospital-centric to a more holistic, integrated EMS system framework. With its global recognition and visibility, the WHO ECSF has the potential to guide the evolution of emergency care systems toward these standards, but it must adapt to advancing knowledge, emerging technologies, and diverse contextual needs. Implementation success will depend on the availability of resources, including funding, data availability, infrastructure, and cultural aspects, necessitating adaptations for local contexts. Future research should focus on evaluating the identified components in terms of process and outcome parameters and assess their applicability across varying global settings.

## Supplementary Information

Supplementary Material 1.

Supplementary Material 2.

## Data Availability

No datasets were generated or analysed during the current study.

## Abbreviations

BLS: Basic Life Support
CPH: Copenhagen
EAB: Emergency Access Button
ED: Emergency Department
EMCC: Emergency Medical Coordination Center
EMCC: Emergency Medical Dispatch Center
EMS: Emergency Medical Services
EMT: Emergency Technicians
GP: General Practitioner
GRA: Global Resuscitation Alliance
ICD-10: International Statistical Classification of Diseases and Related Health Problems, 10th Revision
IH: In-hour
MECU: Physician-manned mobile emergency care unit
1813: Medical Helpline 1813, Copenhagen Health Region
OHCA: Out-of-Hospital Cardiac Arrest
OOH: Out-of-hour
PEMS: Prehospital Emergency Medical Services
WHA: World Health Assembly
WHO ECSF: WHO Emergency Care Systems Framework
