# Supplementary material for: Strengthening the WHO Emergency Care Systems Framework: insights from an integrated, patient-centered approach in the Copenhagen Emergency Medical Services system—a qualitative system analysis
Source: BMC Health Serv Res. 2025 Mar 18;25:401. doi: 10.1186/s12913-025-12465-7 (PMC11916934; doi:10.1186/s12913-025-12465-7)
Supplement: Supplementary file 1 — Supplementary Material 1. [file 12913_2025_12465_MOESM1_ESM.docx]

# **Additional File 1. Literature Search**

**Table 1** List of Potential Search Terms

| Components |  | Process |  | Location |
| --- | --- | --- | --- | --- |
| Emergency Medical Service* OR EMS | AND | Patient Pathway OR Clinical Pathway | AND | Copenhagen |
| Emergency Care |  | Emergency Call |  | Capital Region |
| Prehospital OR pre-hospital |  | Transport OR Transfer |  | Denmark |
| Emergency Department |  | Dispatch* |  | Danish |
| Out of hospital OR OoH OR out-of-hospital |  | Emergency transport |  |  |
| Respond* |  | Bystander |  |  |
| Bystander |  | Health Technology Assessment OR HTA |  |  |
| Paramedic |  |  |  |  |

**Table 2** Database Search Strings

| Database | Access Date | Search Terms | Hits |
| --- | --- | --- | --- |
| PubMed | 14.05. | ((((("Emergency Medical Services"[Mesh]) OR "After-Hours Care"[Mesh]) OR "Ambulances"[Mesh]) OR "Emergency Responders"[Mesh])) AND (Copenhagen OR Danish OR Denmark) AND [Filter: 2011-2021] | 907 |
| Google Scholar | 22.05. | ("emergency medical service" OR "emergency medical services" OR EMS OR dispatch* OR "prehospital" OR "pre-hospital" OR "out-of-hospital" OR "out of hospital") AND (Copenhagen OR Danish) [Filter 2011-2021] | 28,800  (sorted by best match;  21 pages screened: until 10 successive non-relevant hits were obtained in title screening)* |
| *This approach is in line with findings from Haddaway et al. (2015) who indicate that due to the abundance of hits, the screening of the first 5 - 10 pages (50 - 100 hits) may already provide the adequate results especially with regards to grey literature. | | | |

**Table 3** Eligibility Criteria for Literature Selection

| Component | Inclusion Criteria | Exclusion Criteria |
| --- | --- | --- |
| Relevance | Literature containing relevant information on good or best practices at the CPH EMS and records concerning practices at the CPH EMS with an improved health outcome compared to the status quo (e.g. before the intervention). | No mentioning of CPH EMS-related inputs, processes or outcomes. Focusing on patient or workforce characterization or assessment of compliance. |
| Year | Published since 2011 | If published after 2011 but concerns data/organizational processes from before 2011.* |
| Geographical area | Copenhagen, Capital Region of Denmark | Other than Capital region of Denmark |
| Type of Literature | peer-reviewed articles, abstracts, grey literature including websites focusing on healthcare and EMS, reports, legal texts and internal documents from the CPH EMS | None applied. |
| Outcomes | Descriptions of CPH EMS structures and components related to inputs, processes, outcomes; or effectiveness of population-based interventions | Assessment analysis of specific treatment/drug; Descriptive studies with regards to patient/workforce characterization |
| Language | All indexed in English language. | None applied. |
| *Excluded was all literature referring to information before the organizational transformation of the CPH EMS in 2011, as they are no longer relevant in current practices (29,45). | | |

**Table 4** Overview of key components of CPH EMS and potential insights for WHO ECSF of included literature

| Author (Year) | Title | Study Design | Main Findings | Key Components of CPH EMS  (Integrated & Patient-Centered Approach) | Potential Insights for  Enhancing WHO ECSF |
| --- | --- | --- | --- | --- | --- |
| Jensen et al. (2020) | The Copenhagen Tool a research tool for evaluation of basic life support educational interventions | Mixed methods study incl. international expert panel meetings, a modified Delphi consensus process, manikin data and qualitative interviews | Developed and validated the Copenhagen Tool for assessing and comparing BLS education interventions for adults. | The Copenhagen Tool for evaluating BLS educational interventions | Assessment of BLS education effectiveness |
| Jensen et al. (2019) | A nationwide investigation of CPR courses, books, and skill retention | Nationwide survey | Identified inconsistencies in CPR course content across Denmark, with skill retention declining significantly within six months post-training. Recommended standardized training to improve long-term competency. | CPR courses, skill retention, and educational materials | Importance of structured training programs for EMS personnel |
| Andelius et al. (2020) | Smartphone Activation of Citizen Responders to Facilitate Defibrillation in Out-of-Hospital Cardiac Arrest. | Retrospective study from the Stockholm Region of Sweden and the Capital Region of Denmark | Bystander defibrillation was more frequent in both private homes and public spaces when a volunteer responder arrived before EMS, bystander defibrillation was 7 times higher in private homes when volunteer responders arrived before EMS compared with cases where the EMS arrived firs | Smartphone-activated citizen responders for OHCA | Integration of digital solutions to enhance community response |
| Berglund et al. (2018) | A smartphone application for dispatch of lay responders to out-of-hospital cardiac arrests | Prospective study | The smartphone app effectively alerts CPR-trained volunteers to out-of-hospital cardiac arrests, but improvements are needed to reduce the time to defibrillation before EMS arrival. | Lay responders dispatched via smartphone app | Strengthening community involvement in EMS |
| Hansen et al. (2017) | Bystander Defibrillation for Out-of-Hospital Cardiac Arrest in Public vs Residential Locations | Cohort study | Among 18,688 patients with out-of-hospital cardiac arrests (OHCAs), bystander defibrillation significantly increased in public locations from 2001 to 2012, with survival rates improving, while it remained limited in residential locations despite efforts to expand access to AEDs. | Bystander defibrillation in public vs. residential areas | Enhancing AED accessibility and public education strategies |
| Agerskov et al. (2015) | Public Access Defibrillation: Great benefit and potential but infrequently used | Observational study | Thirty-day survival for OHCA with a shockable rhythm was 64% with pre-ambulance AED use versus 47% without. Despite 15.1% of OHCAs occurring within 100m of an AED, only 3.8% had one applied before EMS arrival. | Public access defibrillation | Increased accessibility of AEDs to improve survival rates |
| Hansen et al. (2014) | Systematic downloading and analysis of data from automated external defibrillators used in out-of-hospital cardiac arrest | 20-month, prospective, observational study | Of 121 deployed AEDs, 91 were for presumed cardiac-origin OHCAs. AED records showed a significantely greater prevalence of initial shockable rhythm (55%) compared to the EMS records (27.6%). Shockable cases were more often witnessed, had higher bystander CPR rates, and had a higher 30-day survival rate. | Systematic AED data download and analysis | Standardized post-event data evaluation in EMS |
| Karlsson et al. (2019) | Automated external defibrillator accessibility is crucial for bystander defibrillation and survival: A registry-based study. | Registry-based study | Of 2,500 OHCAs, 22.6% were near a registered AED, but fewer than half were accessible. When an AED was available, bystander defibrillation was three times more likely, and 30-day survival nearly doubled. Accessibility significantly improved outcomes, highlighting the need for better AED availability. | AED accessibility impact on survival outcomes | Optimizing AED placement and accessibility strategies |
| Andersen et al. (2013) | Implementing a nationwide criteria-based emergency medical dispatch system: a register-based follow-up study. | Register-based follow-up study | Most patients were classified as Danish Index emergency level A or B (A: 51,4%; B:46,3%), with level A cases (urgent cases) facing significantly higher fatality and hospital admission risks. It is concluded that the new criteria-based dispatch system effectively prioritizes high-risk patients for the highest emergency level. | Nationwide implementation of criteria-based EMS dispatch | Improved triage protocols to optimize resource allocation |
| Andersen et al. (2011) | Criteria-based emergency medical dispatch of ambulances fulfils goals | Observational cohort study | Confirmed that criteria-based dispatch maintained response efficiency The distribution according to level of urgency was 28.7% for the highest level of urgency (A) , while the largest group, 35.1% of patients, were level D cases (transport but no ambulance needed). Target response time for level A (6,5min.) and B (11,9min) were met. | Criteria-based triage and dispatch and response time monitoring | Enhancing dispatch efficiency through structured criteria |
| Blomberg et al. (2021) | Effect of Machine Learning on Dispatcher Recognition of Out-of-Hospital Cardiac Arrest During Calls to Emergency Medical Services: A Randomized Clinical Trial | RCT | A machine learning model identified 5,242 suspected OHCA cases from 169,049 emergency calls. Dispatchers using AI assistance recognized 93.1% of confirmed OHCAs, compared to 90.5% without it (P = .15). While AI had higher sensitivity (85.0% vs. 77.5%; P < .001), it had lower specificity and predictive value. The trial found no significant improvement in dispatcher recognition with AI support, despite AI outperforming human detection. | Machine learning for OHCA recognition in EMS calls | AI-assisted decision-making to improve emergency response |
| Linderoth et al. (2015) | Challenges in out-of-hospital cardiac arrest - A study combining closed-circuit television (CCTV) and medical emergency calls | CCTV & emergency call analysis | CCTV footage of 21 OHCA incidents revealed challenges in situation awareness, communication, and attitude/approach. Bystanders often had better physical access than callers, yet information had to pass through the caller to them. Many bystanders left, and callers failed to delegate tasks like retrieving an AED. Integrating CCTV with emergency call audio could enhance understanding and improve response, with dispatchers potentially acting as remote team leaders to coordinate resuscitation efforts. | Evaluating challenges in audio-only OHCA response efforts | Identifying barriers to timely intervention through evaluation of the status quo, using CCTV as visual data to broaden data coverage |
| Linderoth et al. (2019) | Medical dispatchers' perception of visual information in real out-of-hospital cardiac arrest: a qualitative interview study | Qualitative interview study | Dispatchers found live video from emergency scenes useful but noted difficulties including logistical issues, delayed dispatch, poor image quality, and information overload, which may cause confusion when focusing on multiple aspects at once. | CCTV footage contributed valuable information about the OHCA patient, the physical setting, and the bystanders’ response | Benefit of visual information during emergency call, especially dispatcher-assisted CPR |
| Ebert et al. (2020) | Does an emergency access button increase the patients' satisfaction and feeling of safety with the out-of-hours health services? A randomised controlled trial in Denmark | Randomised controlled parallel superiority trial using questionnaire survey | Out of 6,704 invited callers, 32.9% (2,208) responded to the questionnaire, with 1,415 in the intervention group (including 621 users and 794 non-users). The EAB option improved satisfaction with waiting times, overall satisfaction, and the feeling of safety. About 72% of EAB users felt their safety was significantly increased, compared to 25% of non-users. The study concludes that the EAB provides quick access to out-of-hours healthcare advice, benefiting those in urgent need and significantly enhancing both safety and satisfaction. | Emergency access button in out-of-hours services | Improving patient access to urgent care |
| Ebert et al. (2017) | Development and evaluation of an "emergency access button" in Danish out-of-hours primary care: a study protocol of a randomized controlled trial. | RCT-Study Protocol | The study aims to develop an "emergency access button" (EAB) that allows patients to bypass the standard waiting line in out-of-hours primary care if they believe their condition is urgent, and to assess its impact on patient satisfaction and their sense of safety. | Need for urgency triage during waiting times  Need to assess patient user behavior | Improved patient access differentiated by urgency and severity |
| Zinger et al. (2019) | Satisfaction of 30 402 callers to a medical helpline of the Emergency Medical Services Copenhagen: a retrospective cohort study | Retrospective cohort study | Of 30,402 callers, 73% were satisfied with the medical helpline. Satisfaction was higher for somatic injuries, face-to-face consultations, or waiting times under 10 minutes. Parents of children aged 0-4 were more satisfied when calling for somatic issues or receiving a phone consultation. | Patient satisfaction with medical helpline services | Evaluating patient experience to refine EMS operations |
| Ebert et al. (2019) | Do callers to out-of-hours care misuse an option to jump the phone queue? | Descriptive study of a randomized intervention | The EAB, a shortcut to jump the OOH waiting line, was used as intended as higher proportions of severe health problems were found among EAB users compared to EAB non-users. | Testing Emergency access button (EAB) usage to bypass telephone queue with perceived severe health problems to bypass the queue | Pre-Triage options in case of telephone waiting line; Understanding patient behavior and use as intended |
| Ebert et al. (2019) | Giving callers the option to bypass the telephone waiting line in out-of-hours services: a comparative intervention study | Comparative intervention study | Patient characteristics associated with increased EAB use were male gender, higher age, low education, being retired, and increasing announced estimated waiting time. Only about 3% of all callers chose to bypass the waiting line in the OOH service when given the option with EAB | Testing user behavior to evaluate effectiveness of EAB intervention | Timely care and bypass options for cases with severe health problems through short waiting times or bypass options |
| Moller et al. (2015) | Why and when citizens call for emergency help: an observational study of 211,193 medical emergency calls | Observational study | Causes for emergency calling were categorized with the most frequent being ”unclear problems”(19 %), followed by medical problems, intoxication and accidents. The majority of calls were assigned the highes9+ priority level. | Analysis of emergency calls regarding causes and emergency priority level. Every fith call fell under the unclear category showing improvement potential of the triage tool or education for calltakers | Data-driven insights into EMS demand and utilization |
| Ersbøll et al. (2017) | Evaluering af Sociolancen | Evaluation study | The Sociolance effectively bridges social and healthcare services by alleviating ambulance and police workloads, improving referrals through expert assessment and relationship-building. | Specialized unit Sociolancen for outreach work with multiprofessional team | Specialized unit for socially vulnerable population |
| Østergaard & Lyngby (2019) | Evaluation of dispatch outcomes and staffing of the Copenhagen mobile health and social care unit –Sociolancen | Evaluation study | Paramedical and social staffing is needed when attending the homeless and socially deprived citizens. With half of dispatches covering outreach work with little referral to emergency ambulances, the Sociolance is believed to be well dispatched. | Copenhagen’s mobile health & social care unit (Sociolance) | Integrating social care into emergency services |
| Alstrup et al. (2019) | The Danish helicopter emergency medical service database: high quality data with great potential | Database study | The national HEMS database with 99% data completeness, facilitates performance monitoring and future research. | Danish HEMS database for critical care transport on mission- and patient-specific data related to the pre-hospital pathway | High-quality data utilization for EMS optimization |
| Alstrup et al. (2019) | Characteristics of patients treated by the Danish Helicopter Emergency Medical Service from 2014-2018: a nationwide population-based study | Nationwide population-based study | The Danish HEMS primarily treats critically ill or injured patients, performing critical care and enabling rapid transport to specialized treatment, especially in rural areas, with cardiovascular, trauma, and neurological emergencies being most common. | Characteristics of HEMS-treated patients | Monitoring of patient population and patient needs |
| Barfod et al. (2012) | Abnormal vital signs are strong predictors for intensive care unit admission and in-hospital mortality in adults triaged in the emergency department - a prospective cohort study | Prospective cohort study | The HAPT system effectively predicts ICU admission and in-hospital mortality, with higher triage urgency linked to increased risk. Key predictors include SpO₂, respiratory rate, systolic BP, and GCS, along with the number of abnormal vital signs. Presenting complaints often indicated greater urgency than vital signs, potentially leading to over-triage. | Vital signs predicting ICU admission & mortality | Refining triage algorithms in emergency settings |
| Hasselbalch et al. (2016) | The Copenhagen Triage Algorithm: a randomized controlled trial | RCT | The Copenhagen Triage Algorithm study is a prospective, two-center, cluster-randomized, cross-over, non-inferiority trial that evaluates CTA against the Danish Emergency Process Triage. | Copenhagen Triage Algorithm | Standardized triage model for emergency care |
| Pedersen et al. (2006) | Patients' satisfaction with the Mobile Emergency Care Unit in Copenhagen | Qualitative questionnaire study | The majority of patients was satisfied with the service provided by the Mobile Emergency Care Unit (MECU) | Patient satisfaction with the MECU | Evaluating patient satisfaction |
| Christiansen et al. (2021) | Patients Own Safety Incidents Reports to the Danish Patient Safety Database Possess a Unique but Underused Learning Potential in Patient Safety | Register-based study | Few safety incidents (1.4%) were reported by patients, but most were accepted and classified in the DPSD. Patient reports were longer, more emotional, and focused on communication and health consequences, while professionals' reports were concise and technical. | Incidence reporting platform also for patients | Monitoring safety incidence to identify system improvement area |
| Moeller et al. (2016) | Learning and feedback from the Danish patient safety incident reporting system can be improved | Qualitative questionnaire study | Gaps in incident learning and feedback mechanisms were identified, highlighting the need for iproved implementation of the DPSD.. | Improving incidence reporting system especially with regards to  implementation of learning processes and feedback mechanisms | Constant review and further development of technical platforms |
| Region Hovedstaden Akutberedskab (2020) | Akutberedskabet Årsrapport 2019 | Report |  | Monitoring performance metrics EMCC and EMS units  Current Research Output | Effects of integrated EMCC and specialized care units |
| Regionernes Kliniske Kvalitetsudviklingsprogram (2021) | Databasen for Akutte Hospitalskontakter - Årsrapport 2019 | Report |  | National quality monitoring of acute patient care in Danish hospitals | Monitoring of acute patient care and geographical benchmarking |
| Regionernes Kliniske Kvalitetsudviklingsprogram (2021) | Års- og aktivitetsrapporter | Report |  | Monitoring performance metrics EMCC and EMS | Monitoring of EMS performance |
| Region Hovedstaden Akutberedskab (2020) | Årsrapport 2020: Nye opgaver, ekstraordinært mange opkald til Akuttelefonen 1813 og god patienttilfredshed | Report |  | Monitoring performance metrics EMCC and changes due to Covid-19 | Monitoring and adaptability to specific situations |
| Region Hovedstaden (2016) | Babyer får ny ambulance | Web Page |  | Report on Babylance use and performance | Specialized unit for pediatric patients |
| Region Hovedstaden Akutberedskab (n.d.) | Physician staffed Critical Care Units | Web Page |  | Report on Physician staffed Critical Care Units and performance | Differentiated EMS response |
| Sundheds- og Ældreministeriet (2016) | Bekendtgørelse om planlægning af sundhedsberedskabet | Legal Text |  | Legal definition and responsibilities of health emergency preparedness planning | Legal framework to enable needs-based emergency care |
