# Supplementary material for: Strengthening the WHO Emergency Care Systems Framework: insights from an integrated, patient-centered approach in the Copenhagen Emergency Medical Services system—a qualitative system analysis
Source: BMC Health Serv Res. 2025 Mar 18;25:401. doi: 10.1186/s12913-025-12465-7 (PMC11916934; doi:10.1186/s12913-025-12465-7)
Supplement: Supplementary file 2 — Supplementary Material 2. [file 12913_2025_12465_MOESM2_ESM.docx]

**Additional File 2. Expert Interviews**

1. **Selection of Experts**

To supplement published literature on the Copenhagen EMS (CPH EMS), expert interviews were conducted. The aim of the selection process was to assign at least one expert for each component of the WHO Emergency Care Systems Framework (WHO ECSF). This aim was met.

Experts were identified through the following three paths (figure 1):

1. As authors of a relevant publication in peer-reviewed articles on the CPH EMS,
2. Recommended by a senior-level executive and a senior-level researcher at the CPH EMS, and/or
3. Through recommendation of already interviewed experts.

A preliminary list of experts was then verified by a senior-level executive and a senior-level researcher. Verification means the assessment of the (a) suitability of the prospective interviewee’s expertise with respect to the intended component of the WHO ECSF, and (b) further suggestions for suitable experts. This was especially useful to identify working professionals as experts that had not published in a peer-reviewed journal.


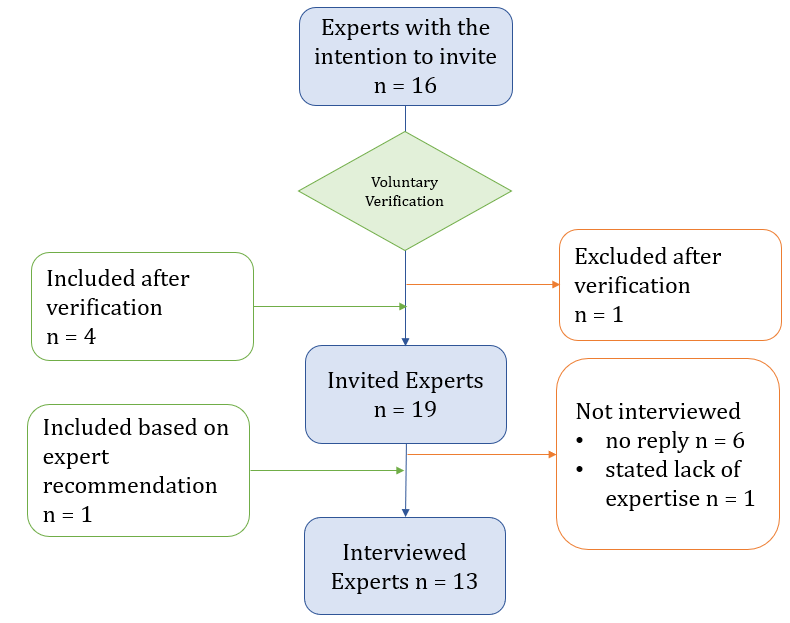


**Figure 1.** Flowchart of Expert Selection.

All interviewed experts signed consent forms for the processing of personal data, the audiotaping and pseudnoymized publication. The duration of each interview was approximately 30 minutes. Only the researcher and interviewee were present at the interview. No repeat interviews were carried out. The semi-structured interview was designed as follows:

1. **Design of semi-structured questionnaires**

Three-part semi-structured questionnaires with open-ended questions were developed based on:

1. Domains and questions based on the prehospital EMS assessment tool by Mehmood et al. (2018) covering the prehospital EMS Framework which is based on the WHO Health System Building Blocks,
2. questions directly related to the WHO ECSF, and
3. individualized questions^[[1]](#footnote-1)^ based on the expert’s publications or current research projects with focus on the experts’ perceived areas of innovation.

1. All thirteen questionnaires were partially individualized to the respective expert’s area of research or current profession to ensure fitting of the questions to the interviewees field of expertise. This approach allowed in-depth questions while covering all important domains of EMS systems according to the WHO ECSF. Moreover, it provided insights into practical knowledge, as currently little peer-reviewed information on the functioning of the CPH EMS system were published in recent years. [↑](#footnote-ref-1)
